# Neuroimaging and machine learning for brain age estimation

**DOI:** 10.18632/aging.204694

**Published:** 2023-04-28

**Authors:** Thomas D. Kocar, Michael Denkinger, Jan Kassubek

**Affiliations:** 1 Geriatric Center Ulm, University of Ulm at Agaplesion Bethesda Ulm, Germany

**Keywords:** brain age, machine learning, neuroimaging, magnetic resonance imaging, neurodegeneration

The morphological correlates of brain aging in neuroimaging are characterized *grosso modo* by a decline in gray matter volume and a reduction in white matter integrity - these changes are associated with cognitive decline including memory, attention, and executive functions and are thought to be the result of a combination of factors from predisposition and environment. There is converging evidence that regionally differential aging processes in the human brain exist, mainly affecting the frontal lobe and relatively sparing posterior and infratentorial areas [1]. Magnetic Resonance Imaging (MRI) is capable of providing high-resolution images of the brain that can be assessed for age-related changes [2]. Machine learning (ML), as an emerging artificial intelligence (AI)-based field in medicine, has been applied to quantify these MRI changes in the human brain, commonly accomplished by (1) extracting features from brain images and (2) training an ML model to predict the age from the extracted features [3]. Application to (advanced) MRI data obtains an age estimation that is considered to be representative of an individual’s brain age/health. The difference between estimated brain age and chronological age is called the brain age gap (BrainGAP) or brain predicted age difference (Brain-PAD) and is thought to potentially serve as a biomarker for processes like accelerated/delayed brain aging. The BrainGAP was shown to correlate with other imaging biomarkers of brain aging, such as white matter hyperintensities [4], and appears to be related to cardiovascular risk factors associated with accelerated aging in general, such as systolic/diastolic blood pressure, smoking habits, and cardiac function.

Although the performance of most brain age estimators is promising [3, 5], a major challenge in moving to a clinical application is the lack of an automated, scanner-independent data preprocessing pipeline as well as robust and generalizable ML models that work with any MRI data type [5]. Diffusion tensor imaging (DTI) as an established MRI modality to map structural brain white matter connectivity is one first-line candidate: diffusivity can be robustly measured across different MRI scanners and field strengths and different tract systems of the brain have been shown to demonstrate differential alterations during (healthy) aging [6]. Proof-of-concept ML studies have already demonstrated the validity of DTI in single modality approaches for brain age estimation [7]. In the technical domain, data preprocessing and feature extraction are generally required to prepare DTI data for ML. For this purpose, diffusion metrics are usually aggregated at the tract level. Multiple ways to automate tract segmentation have been proposed, such as tract-based spatial statistics, fiber tracking, and U-Nets. Recently, a large-scale application of tract segmentation was demonstrated using the UK Biobank imaging dataset, paving the way for multisite and finally transnational DTI data analyses [8].

For brain age estimation, artificial neural networks (ANN) have been established as very promising ML models due to their superior performance in solving complex non-linear problems. ANN are capable of grasping complex interactions of different white matter tracts which are important for assessing brain health. Although multimodal ML models perform best, single MRI modality approaches may be a better candidate to achieve widespread clinical adoption, where ease of use is important. In addition, convolutional neural networks and other deep learning algorithms applied to conventional T1w images have the potential to match DTI in its predictive power [3]; however, it remains to be explored to what extent these algorithms can be made scanner-agnostic.

In a recent study on brain aging, an ANN was capable of estimating the subjects’ chronological age with high accuracy by solely relying on DTI data [7]. In addition to brain age estimation, the ANN could also perform the inverse operation, i.e., age correction, which was done by modifying the gradient descent algorithm to alter the input data [7]. The proposed age correction algorithm might be extended to create synthetic data and possibly aid in data augmentation, which is important for training large-scale ML models and for studying rare diseases. Although still in its infancy, ANN age correction algorithms may increase diagnostic accuracy by separating “normal” brain aging (i.e., without a recognized pathological process) from degenerative, vascular, inflammatory, and other diseased conditions. (As a side note: possibly, the term “normal” or “healthy” aging of the brain might receive a new definition by this approach.) For that purpose, age-corrected models could be applied to large, longitudinal datasets with well-defined and verified pathologies. Using longitudinal data, healthy people at baseline can be further distinguished from those with already subclinical prodromal diseases and pathological aging with early signs of prefrailty and frailty.

Beyond these developments in brain aging research, recent advances in ML have exceeded the expectations of many AI experts, with transformer-based deep learning models leading the field. In the resources-intensive field of geriatrics, automation and intelligent systems are the keys to mastering the challenges of demographic change in societies worldwide. In this environment, the application of AI to neuroimaging targeted at aging in particular is well positioned to improve and expand geriatric diagnostics in the future.
